# Mesenchymal stem cell-derived extracellular vesicles for kidney repair: current status and looming challenges

**DOI:** 10.1186/s13287-017-0727-7

**Published:** 2017-12-04

**Authors:** Arash Aghajani Nargesi, Lilach O. Lerman, Alfonso Eirin

**Affiliations:** Division of Nephrology and Hypertension, Mayo Clinic, 200 First Street SW, Rochester, MN 55905 USA

**Keywords:** Mesenchymal stem cells, Extracellular vesicles, Microvesicles, Exosomes, Kidney

## Abstract

Novel therapies are urgently needed to address the rising incidence and prevalence of acute kidney injury (AKI) and chronic kidney disease (CKD). Mesenchymal stem/stromal cells (MSCs) have shown promising results in experimental AKI and CKD, and have been used in the clinic for more than a decade with an excellent safety profile. The regenerative effects of MSCs do not rely on their differentiation and ability to replace damaged tissues, but are primarily mediated by the paracrine release of factors, including extracellular vesicles (EVs), composed of microvesicles and exosomes. MSC-derived EVs contain genetic and protein material that upon transferring to recipient cells can activate several repair mechanisms to ameliorate renal injury. Recent studies have shown that MSC-derived EV therapy improved renal outcomes in several animal models of AKI and CKD, including ischemia-reperfusion injury, drug/toxin-induced nephropathy, renovascular disease, ureteral obstruction, and subtotal nephrectomy. However, data about the renoprotective effects of EV therapy in patients with renal failure are scarce. This review summarizes current knowledge of MSC-derived EV therapy in experimental AKI and CKD, and discusses the challenges that need to be addressed in order to consider MSC-derived EVs as a realistic clinical tool to treat patients with these conditions.

## Background

Kidney disease is a prominent challenge for health care systems. Incidence and mortality rates of both acute kidney injury (AKI) and chronic kidney disease (CKD) have increased in recent decades [1]. It is estimated that during a hospital admission one in five adults and one in three children experience AKI, a sudden episode of kidney failure or kidney damage [2]. CKD, a condition characterized by a gradual loss of kidney function, is estimated to be quite prevalent. In the US alone, its predicted prevalence rate is 13.6%, with more than 670,000 patients in end-stage renal disease (ESRD) [3, 4], the final stage of CKD when irreversible loss of renal function mandates dialysis or kidney transplantation. Both AKI and CKD consume considerable healthcare resources and are associated with significant economic costs. AKI is responsible for more than 5% of overall hospital expenses [5], and more than $80 billion of the Medicare budget is spent to care for CKD and ESRD patients, accounting for over 18% of its total expenditure [4, 6]. AKI can cause ESRD directly, and increase the risk of developing CKD and worsening of underlying CKD [7]. Importantly, AKI and CKD are risk factors for developing cardiovascular disease and mortality [8]. Therefore, the rising incidence and prevalence of AKI and CKD and their deleterious complications underscore the need to identify more effective therapeutic strategies to attenuate renal injury and prevent its progression to ESRD.

Mesenchymal stem/stromal cells (MSCs) are multipotent cells with robust self-renewal, regenerative, proliferative, and multi-lineage differentiation potential [9]. By definition, MSCs are characterized by the expression of MSC markers and the ability to differentiate into adipocytes, chondrocytes, and osteocytes [10]. Emerging evidence supports the existence of kidney-resident MSCs, which originate from renal pericytes that form an extensive network around the microvasculature [11]. Although the entire spectrum of their function still remains to be elucidated, they play key roles in regulation of renal blood flow, capillary permeability, endothelial survival, and immunologic surveillance [12]. In addition, MSCs with potent proangiogenic and immunomodulatory properties can also be isolated from various extrarenal sources, including adipose tissue, making them ideal candidates for renal regenerative therapy [13, 14].

According to ClinicalTrials.gov there are currently 46 ongoing or completed clinical trials using MSC therapy for AKI and CKD, including diabetic nephropathy, focal segmental glomerulosclerosis, systemic lupus erythematous, and kidney transplantation [15–17] (Table 1). In an ongoing phase I clinical trial, patients with cisplatin-induced AKI and solid organ cancer are followed for 1 month after a single systemic infusion of allogeneic bone marrow-derived MSCs (NCT01275612). Primary and secondary end points include the rate of decline in renal function and urinary injury markers, respectively. Cardiac surgery patients at high risk of postoperative AKI were treated safely with allogeneic MSCs [18, 19]. Systemic administration of autologous bone marrow-derived MSCs in patients with autosomal dominant polycystic kidney disease did not cause any serious adverse events and decreased serum creatinine levels after 12 months of follow-up [20]. Preliminary results of a randomized clinical trial in patients with diabetic nephropathy also showed stabilized or improved glomerular filtration rate (GFR) after 3 months of treatment with allogenic MSCs [21]. Likewise, intra-arterial infusion of autologous MSCs in patients with renovascular disease (RVD) increased cortical perfusion and renal blood flow (RBF), and reduced renal tissue hypoxia in the post-stenotic kidney [22]. Clinical trials are also testing the immunomodulatory and renoprotective properties of MSCs after renal transplantation (NCT02409940). Autologous MSCs were found to be superior to conventional immunosuppressive therapy in preventing acute rejection, decreasing opportunistic infections, and preserving renal function in patients undergoing renal transplant [23]. Taken together, these studies indicate that MSC therapy is safe, feasible, well tolerated, and effectively ameliorates renal pathology in a wide range of diseases.

**Table 1:** Clinical studies testing the efficacy of MSCs in AKI and CKD

Condition	ID	Title	Link	Status
AKI	NCT01275612	Mesenchymal stem cells in cisplatin-induced acute renal failure in patients with solid organ cancers	https://clinicaltrials.gov/ct2/show/NCT01275612	Recruiting
	NCT00733876	Allogeneic multipotent stromal cell treatment for acute kidney injury following cardiac surgery	https://clinicaltrials.gov/ct2/show/NCT00733876	Completed
	NCT01602328	A study to evaluate the safety and efficacy of AC607 for the treatment of kidney injury in cardiac surgery subjects	https://clinicaltrials.gov/ct2/show/NCT01602328	Terminated
CKD	NCT02166489	Mesenchymal stem cells transplantation in patients with chronic renal failure due to polycystic kidney disease	https://clinicaltrials.gov/ct2/show/NCT02166489	Completed
	NCT01843387	Safety and efficacy of mesenchymal precursor cells in diabetic nephropathy	https://clinicaltrials.gov/ct2/show/NCT01843387	Completed
	NCT02266394	Hypoxia and inflammatory injury in human renovascular hypertension	https://clinicaltrials.gov/ct2/show/NCT02266394	Recruiting
	NCT02409940	To elucidate the effect of mesenchymal stem cells on the T-cell repertoire of the kidney transplant patients	https://clinicaltrials.gov/ct2/show/NCT02409940	Ongoing
	NCT00658073	Induction therapy with autologous mesenchymal stem cells for kidney allografts	https://clinicaltrials.gov/ct2/show/NCT00658073	Completed

*AKI* acute kidney injury, *CKD* chronic kidney disease

**Fig. 1:**
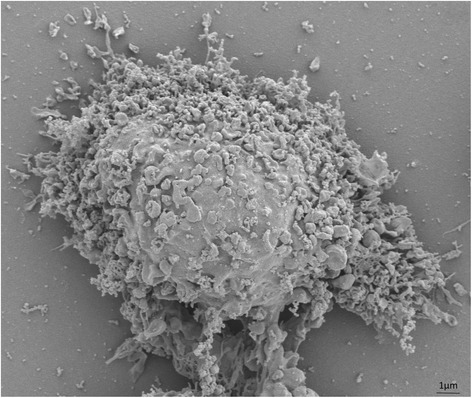
Scanning electron microscopy image showing a cultured porcine adipose tissue mesenchymal stem cell releasing extracellular vesicles. This figure is original for this article

Mounting evidence supports the notion that MSCs exert their reparative effects by releasing extracellular vesicles (EVs), including exosomes with a diameter of 30–120 nm, and micro-vesicles ranging from 100 nm to 1 μm in size [24]. Exosomes arise form endocytic compartments, known as microvesicular bodies, and are released into extracellular space through fusion with plasma membrane [25]. In contrast, microvesicles originate from outward buddings of cell membrane and their release is controlled by calcium influx and cytoskeletal reorganization, among several other factors [25]. We have previously shown that porcine MSCs release EVs (Fig. 1) that are selectively packed with proteins, mRNAs, and microRNAs [26–28]. Furthermore, we recently proposed that genes, proteins, and microRNAs enriched in EVs have the potential to modulate selective cellular pathways in recipient cells [29]. Therefore, MSC-derived EVs may exert trophic and reparative effects, representing an attractive non-cellular approach for treating renal disease. Indeed, recent studies have shown that delivery of MSC-derived EVs is safe and can improve kidney function in several models of AKI and CKD. The purpose of this review is to summarize the current knowledge of MSC-derived EV therapy in experimental AKI and CKD, and discusses the challenges that need to be addressed in order to consider MSC-derived EVs as a realistic clinical tool to treat patients with these conditions.

## MSC-derived EVs in experimental AKI

### Ischemia-reperfusion injury

Renal ischemia-reperfusion injury (IRI), a condition caused by initial sudden cessation of blood flow to the kidney followed by restoration of blood flow and re-oxygenation, is one of the primary causes of AKI associated with significant morbidity and mortality [30]. Although the pathophysiology of renal IRI remains obscure, both hypoxia at ischemic phase and subsequent generation of reactive oxygen species at reperfusion initiate a cascade of deleterious responses characterized by inflammation and cell death that subsequently leads to AKI [31]. A number of studies have recently tested the efficacy of MSC-derived EVs to blunt experimental IRI-induced AKI (Table 2). Lindoso et al. [32] tested the biological effect of EVs in an in vitro model of renal IRI induced by ATP depletion of tubular cells, which were subsequently co-incubated with MSC-derived EVs. EVs progressively incorporated into damaged tubular cells, suggesting higher uptake under stressful conditions. EVs decreased cell death and restored proliferation of ATP-depleted tubular cells. This was paralleled with downregulated expression of a specific set of microRNAs involved in apoptosis, hypoxia, and cytoskeletal reorganization, suggesting that EVs can protect tubular cells against metabolic stress by mechanisms involving post-transcriptional regulation.

**Table 2:** Experimental studies testing the efficacy of MSC-derived EVs in IRI-AKI

Type of model	Species	Intervention	Administration methods	Main findings	Reference
In vitro, tubular epithelial cells	-	Human bone marrow MSC-derived EVs	Incubation in culture media	• EVs incorporated into injured cells • Downregulated miRNAs associated with apoptosis, cytoskeleton and hypoxia • Downregulated microRNAs involved in apoptosis, fibrosis, hypoxia, and cytoskeletal reorganization	Lindoso et al. 2014 [32]
In vivo	Rat	Human bone marrow MSC-derived EVs	Intravenous	• EVs decreased tubular injury and apoptosis • Improved cell proliferation and renal function • Transferred RNA-based information to recipient cells	Gatti et al. 2011 [33]
In vivo	Rat	Autologous bone marrow MSC-derived EVs	Intravenous	• EVs decreased tubular injury, apoptosis, and inflammation • Improved renal function	Wang et al. 2014 [34]
In vivo	Rat	Human umbilical cord MSC-derived EVs	Intravenous	• EVs decreased renal oxidative stress • Increased renal cell proliferation, attenuated apoptosis and fibrosis, and normalized renal function	Zhang et al. 2014 [35]
In vivo; in vitro, tubular epithelial cells	Rat	Human umbilical cord MSC-derived EVs	Intravenous; incubation in culture media	• EVs improved renal function • Decreased tubular injury, oxidative stress, apoptosis, and necrosis	Zhang et al. 2016 [36]
In vivo	Rat	Human umbilical cord MSC-derived EVs	Intravenous	• EVs reduced apoptosis and enhanced tubular cell proliferation • Improved renal function and ameliorated tubular injury and fibrosis • Increased renal angiogenesis • Transferred proangiogenic-related VEGF and mRNAs to recipient cells	Zou et al. 2016 [37]
In vivo; in vitro, tubular epithelial cells	Rat	Human umbilical cord MSC-derived EVs	Intravenous; incubation in culture media	• EVs upregulated proangiogenic factors • Decreased tubular cell apoptosis, collagen deposition, and fibrosis	Ju et al. 2015 [38]
In vivo; in vitro, umbilical vein endothelial cells	Mouse	Allogenic kidney resident MSC-derived EVs	Intravenous; incubation in culture media	• EVs incorporated into endothelial cells, decreased apoptosis, and increased proliferation and tube formation • Selectively engrafted into injured cells and improved renal function • Ameliorated peritubular capillary rarefaction and improved endothelial cell proliferation	Choi et al. 2014 [39]
In vivo	Rat	Human umbilical cord MSC-derived EVs	Intravenous	• EVs increased renal proliferation • Decreased renal inflammation, tubular and glomerular injury, vascular damage, apoptosis, and fibrosis • Preserved renal function	Zou et al. 2014 [40]
In vivo	Rat	Allogenic adipose tissue MSC-derived EVs	Intravenous	• EVs increased renal angiogenesis and decreased inflammation, oxidative stress, apoptosis, fibrosis • Improved renal function	Lin et al. 2016 [41]
Ex vivo model of renal ischemia, post-circulatory death and pre-transplant	Rat	Allogenic bone marrow MSC-derived EVs	Incubation in buffering solution of donated kidney	• EVs decreased global ischemic damage • Preserved cellular metabolism and viability	Gregorini et al. 2017 [42]

*AKI* acute kidney injury*, EV* extracellular vesicle*, IRI* ischemia-reperfusion injury*, MSC* mesenchymal stem cell*, VEGF* vascular endothelial growth factor

The renoprotective effects of MSC-derived EVs have also been investigated in several in vivo models of renal IRI. In rats subjected to unilateral nephrectomy and renal artery occlusion for 45 min, intravenous MSC-derived EVs immediately after ischemia significantly reduced epithelial tubular cell damage and apoptosis and enhanced their proliferation, improving renal function [33]. Interestingly, the beneficial effect of EVs was mediated in part by the transfer of RNA-based information to recipient cells. Similarly, in rats with renal IRI systemic administration of autologous bone marrow MSC-derived EVs decreased renal injury and improved function, extending the benefits of EVs to ameliorate IRI-induced renal damage and contribute to cellular repair in vivo [34].

EVs harvested from human umbilical cord MSCs have also shown renoprotective benefits in rats with IRI. Intravenous delivery of EVs immediately after the ischemic phase of IRI mitigated renal oxidative damage by decreasing the expression of the pro-oxidant NADPH oxidase-2 [35]. MSC-derived EV-induced attenuation of renal oxidative stress was associated with enhanced renal cell proliferation, decreased apoptosis, and normalized serum creatinine levels 2 weeks after the ischemic insult. Consistent with these findings, intravenous injection of EVs isolated from the conditioned medium of human umbilical cord MSCs after unilateral renal ischemia preserved kidney function and decreased serum levels of the AKI marker neutrophil gelatinase-associated lipocalin [36]. EVs also decreased renal expression of nuclear factor E2-related factor-2, a transcription factor that modulates cellular oxidative stress, which in turn resulted in decreased tubular damage.

Studies in experimental renal IRI have also shown that MSC-derived EVs exert renoprotection by modulating renal angiogenesis. Systemic administration of MSC-derived EVs in rats with renal IRI increased renal capillary density and reduced fibrosis by direct transfer of the proangiogenic factor vascular endothelial growth factor (VEGF) and mRNAs involved in this process [37]. In a similar study, delivery of EVs in rats with IRI increased gene and protein expression of the proangiogenic hepatocyte growth factor, associated with decreased tubular fibrosis [38]. Interestingly, the renoprotective effects of EVs were abolished when EVs were pretreated with RNase, implying that mRNA transfer of proangiogenic factors mediated EV-induced renal repair. The proangiogenic effects of EVs were not limited to those isolated from umbilical cord MSCs. EVs isolated from kidney resident MSCs have been shown to contain several proangiogenic genes, including VEGF, basic fibroblast growth factor, and insulin-like growth factor (IGF)-1 [39]. Systemic administration of allogeneic kidney-resident MSC-derived EVs into mice with renal IRI was followed by engraftment in ischemic kidneys and improvement in renal function, suggesting that delivery of proangiogenic transcripts may contribute to EV-induced renal repair.

Furthermore, administration of MSC-derived EVs has been proved to ameliorate the inflammation that follows IRI. Intravenous delivery of EVs following unilateral renal ischemia in rats decreased the number of kidney macrophages and the expression of the macrophage chemo-attractant factor chemokine C-X-C motif ligand-1 (CXCL1), possibly by transferring into recipient cells microRNAs capable of modulating CXCL1 expression [40]. This treatment boosted tubular proliferation, attenuated fibrosis, and preserved kidney function. Likewise, in rats with IRI induced by bilateral renal artery occlusion and reperfusion, treatment with intravenous MSCs or their EV progeny decreased expression of inflammatory cytokines, including tumor necrosis factor-alpha (TNF-α) and interleukin (IL)-1-β [41]. Combined MSC and MSC-derived EV therapy resulted in an additive effect on amelioration of tubular injury, extending their value to preserve the kidney when delivered in conjunction with MSCs.

MSC-derived EVs may also confer protection against IRI that occurs in kidney donation after circulatory death, preserving renal function prior to kidney transplantation. In a recent study, incubation of donated kidneys with EVs in buffering solution after harvest and prior to transplant decreased ischemic damage by altering the expression of genes encoding enzymes known to improve cell energy metabolism and ion transport [42]. However, it remains to be determined whether the renoprotective effect of MSC-derived EVs is confined to a specific cell type or may prolong graft survival after kidney transplantation. Therefore, these studies suggest that the beneficial effect of MSC-derived EVs in renal IRI is attributable to their antioxidant, immunomodulatory, and proangiogenic properties, and their ability to modulate cell metabolism and several cellular pathways.

### Drug-induced nephropathy

Drug-induced nephropathy (DIN) is a common etiology of AKI that accounts for as high as 60% of both community- and hospital-acquired episodes [43]. Non-steroidal anti-inflammatory drugs, antibiotics, angiotensin converting enzyme inhibitors, and contrast agents have been associated with renal cell toxicity, and may compromise renal function by promoting tubulo-interstitial nephritis and altering intra-glomerular hemodynamics [44]. Recently, the efficacy of MSC-derived EVs has been tested in models of DIN (Table 3). Co-incubation of cisplatin-damaged tubular cells with MSC-derived EVs increased cell proliferation, partly by transferring IGF-1 and IGF receptor-1 [45]. These observations were supported by in vivo studies in animal models of DIN, in which delivery of MSC-derived EVs prevented tubular cell death and enhanced proliferation. For example, administration of MSC-derived EVs into the renal capsule of rats with cisplatin-induced AKI attenuated renal injury and dysfunction partly by reducing formation of pro-oxidants and suppressing activation of pro-apoptotic pathways [46]. Likewise, in mice after cisplatin-induced [47] and glycerol-induced AKI [48, 49] single and multiple intravenous administration of MSC-derived EVs ameliorated tubular injury and improved kidney function. Modulation of apoptosis was implicated in EV-induced renoprotection, which was abolished after degradation of EV mRNA content, suggesting that anti-apoptotic genes shuttled by EVs are the final effectors of their biologic actions.

**Table 3:** Experimental studies testing the efficacy of MSC-derived EVs in DIN-AKI

Type of model	Species	Intervention	Administration method	Main findings	Reference
In vitro, tubular epithelial cells	Mouse	Human bone marrow MSC-derived EVs	Incubation in culture media	•EVs increased cell proliferation •Transferred IGF-1 and IGF-1 receptor	Tomasoni et al. 2013 [45]
In vivo model of cisplatin-induced AKI; in vitro, tubular epithelial cells	Rat	Human umbilical cord MSC-derived EVs	Intra-capsular; incubation in culture media	•EVs attenuated tubular injury, apoptosis, oxidative stress, and necrosis •Improved renal function	Zhou et al. 2013 [46]
In vivo model of cisplatin-induced AKI; in vitro, tubular epithelial cells	Mouse	Human bone marrow MSC-derived EVs	Intravenous; incubation in culture media	•EVs preserved renal structure and function •Decreased renal cell apoptosis	Bruno et al. 2012 [47]
In vivo model of glycerol-induced AKI; in vitro, tubular epithelial cells	Mouse	Human bone marrow MSC-derived EVs	Intravenous; incubation in culture media	•EVs improved renal function •Stimulated tubular cell proliferation and resistance to tubular cell apoptosis •Transferred mRNAs that control transcription, proliferation, and immunoregulation	Bruno et al. 2009 [48]
In vivo model of glycerol-induced AKI; in vitro, tubular epithelial cells	Mouse	Human bone marrow MSC-derived EVs	Intravenous; incubation in culture media	•EVs increased tubular proliferation, prevented necrosis, and preserved renal function • Exosomes and microvesicles with different molecular composition exhibited distinct renoprotective effects	Bruno et al. 2017 [49]
In vivo model of gentamycin-induced AKI	Rat	Autologous bone marrow MSC-derived EVs	Intravenous	•EVs prevented renal dysfunction, necrosis, apoptosis, and inflammation, and increased cell proliferation	Reis et al. 2012 [50]
In vivo model of glycerol-induced AKI	Rat	Human bone marrow MSC-derived EVs	Intravenous	•EVs downregulated genes involved in inflammation, matrix receptor interaction, and cell adhesion molecules •EVs with downregulated miRNAs were ineffective	Collino et al. 2015 [51]
In vivo model of cisplatin-induced AKI; in vitro, tubular epithelial cells	Rat	Human umbilical cord MSC-derived EVs	Intra-capsular	• EVs inhibited apoptosis and inflammation • Activated autophagy, which partly mediated EV renoprotective effects	Wang et al. 2017 [52]

*AKI* acute kidney injury, *DIN* drug-induced nephropathy, *EV* extracellular vesicle, *IGF* insulin growth factor, *MSC* mesenchymal stem cells

Modulation of renal inflammation is an important mechanism by which MSC-derived EVs protect the kidney from toxic drug injury. In rats with gentamycin-induced AKI, EV delivery preserved renal function by preventing the rise in several pro-inflammatory cytokines, including IL-6 and TNF-α, whereas levels of the anti-inflammatory cytokine IL-10 were restored in EV-treated animals [50]. In line with this observation, in mice with glycerol-induced AKI, EV delivery was associated with downregulation of pro-inflammatory genes [51]. However, these studies did not explore whether renal parenchymal or infiltrating inflammatory cells were direct targets of the immunomodulatory effects of EVs. Interestingly, both studies reported that renoprotective effects of MSC-derived EVs were blunted in mice treated with RNA depleted EVs, suggesting an important role for mRNA and/or microRNA shuttling in mediating EV-induced renal recovery after AKI. In line with this notion, a recent study suggested that the anti-apoptotic and immunomodulatory effects of MSC-derived EVs in DIN-AKI are partly mediated by their ability to transfer genes that activate autophagy [52]. Authors found that administration of MSC-derived EVs in the renal capsule of rats with cisplatin-induced AKI increased renal expression of several autophagy-related genes and improved renal function. Taken together, these results indicate that EVs are capable of modulating several pathways involved in the pathogenesis of DIN, and may serve as a novel therapeutic approach in these patients.

## MSC-derived EVs in experimental CKD

### Renovascular disease

Renovascular disease (RVD) is an important cause of secondary hypertension and ESRD in the elderly population [53]. RVD frequently coexists with metabolic syndrome (MetS), a constellation of cardiovascular risk factors that accentuates renal injury and is associated with poor renal outcomes [54]. Recently, our group took advantage of a novel porcine model of coexisting MetS and RVD (MetS + RVD) to test whether intrarenal delivery of autologous MSC-derived EVs would ameliorate structural and functional decline in MetS + RVD kidney [55]. MetS was induced by feeding pigs a high fat/high fructose diet for 16 weeks, whereas RVD was achieved by placing an irritant coil in the main renal artery. We found that a single intrarenal administration of MSC-derived EVs in these pigs attenuated renal inflammation, disclosed by decreased renal vein levels of several pro-inflammatory cytokines, including TNF-α, IL-6, and IL-1-β. Contrarily, renal vein levels of IL-10 increased in EV-treated pigs, associated with a shift from pro-inflammatory to reparative macrophages populating the stenotic kidney, underscoring the immunomodulatory potential of EVs. EVs also improved medullary oxygenation and fibrosis, and restored RBF and GFR, yet animals treated with IL-10 knock-down EVs showed limited renal recovery, implying that this cytokine mediates at least part of their protective effects (Table 4).

**Table 4:** Experimental studies testing the efficacy of MSC-derived EVs RVD-CKD

Type of model	Species	Intervention	Administration method	Main findings	Reference
In vivo model of coexisting metabolic syndrome and RVD	Pig	Autologous adipose tissue MSC-derived EVs	Intrarenal	• EVs decreased renal inflammation • Improved medullary oxygenation and fibrosis, and restored renal blood flow and glomerular filtration rate • Renoprotective effects were partly mediated by IL-10	Eirin et al. 2017 [55]

*CKD* chronic kidney disease, *EV* extracellular vesicle, *MSC* mesenchymal stem cell, *RVD* renovascular disease

### Unilateral ureteral obstruction

Although complete ureteral obstruction is not a common cause of human renal disease, the unilateral ureteral obstruction (UUO) model, which promotes renal parenchymal inflammation, apoptosis, and fibrosis, offers a unique opportunity to study mechanisms responsible for kidney injury [56]. Lately, studies in mouse models of UUO achieved by unilateral ureteral ligation have tested the efficacy of MSC-derived EVs in preventing renal injury (Table 5). Intravenous administration of MSC-derived EVs mitigated tubular injury and fibrosis and improved renal function 2 weeks after UUO [57]. EVs transferred microRNAs capable of modulating fibrosis and epithelial to mesenchymal transition (EMT). In agreement, in vitro experiments in tubular cells treated with the pro-fibrotic transforming growth factor (TGF)-β1 showed that co-incubation with kidney-resident MSC-derived EVs reversed EMT and TGF-β1-induced morphological changes. This mechanism was also confirmed by another study on TGF-β1-treated endothelial cells, in which MSC-derived EVs ameliorated endothelial to mesenchymal transformation and improved cell proliferation 7 days after UUO [58]. Therefore, these studies underscore important anti-fibrotic and renoprotective properties of MSC-derived EVs in experimental UUO.

**Table 5:** Experimental studies testing the efficacy of MSC-derived EVs in UUO-CKD

Type of model	Species	Intervention	Administration methods	Main findings	Reference
In vivo; in vitro, tubular epithelial cells	Mouse	Allogenic bone marrow MSC-derived EVs	Intravenous	• EVs preserved renal function • Decreased tubular injury and epithelial to mesenchymal transition	He et al. 2015 [57]
In vivo; in vitro; human umbilical vein endothelial cells	Mouse	Allogenic kidney MSC-derived EVs	Intravenous	• EVs ameliorated endothelial to mesenchymal transition and improved proliferation • Prevented inflammatory cell infiltration, enhanced proliferation of tubular cells, and decreased apoptosis and microvascular rarefaction	Choi et al. 2015 [58]

*CKD* chronic kidney disease, *EV* extracellular vesicle, *MSC* mesenchymal stem cell, *UUO* unilateral ureteral obstruction

### Subtotal nephrectomy

The renoprotective effects of MSC-derived EVs were also studied in a mouse model of subtotal nephrectomy (STN; Table 6), one of the most widely used experimental models of CKD which is characterized by progressive loss of renal mass and deteriorating renal function [59]. STN was induced by removing one kidney and resecting 5/6 of upper and lower poles of the remaining kidney. Delivery of EVs into the mouse caudal vein 2 days after STN mitigated lymphocyte infiltration and prevented tubular atrophy and fibrosis within 1 week after treatment [60]. Decreased proteinuria, serum creatinine, blood urea nitrogen (BUN), and uric acid levels underscored the potential of MSC-derived EV delivery in preserving the remaining renal function.

**Table 6:** Experimental studies testing the efficacy of MSC-derived EVs in STN-CKD

Type of model	Species	Intervention	Administration method	Main findings	Reference
In vivo	Mouse	Allogenic bone marrow MSC-derived EVs	Intravenous	• EVs improved renal function • Decreased renal fibrosis, inflammation, and tubular atrophy	He et al. 2012 [60]

*CKD* chronic kidney disease, *EV* extracellular vesicle, *MSC* mesenchymal stem cell, *STN* subtotal nephrectomy

## Challenges of MSC-derived EV delivery in human CKD

As discussed above, several studies in animal models of AKI and CKD suggest that MSC-derived EVs can effectively preserve renal structure and function. So far, however, only one clinical trial has tested the renoprotective effects of MSC-derived EVs on the progression of CKD [61]. In this phase II/III pilot study, 40 patients with estimated GFR (eGFR) between 15 and 60 ml/min were randomized to receive either placebo or EVs derived from allogenic cord blood MSCs. Patients were treated with two doses of EVs and followed for 12 months. EV therapy improved eGFR, serum creatinine, and BUN levels, as well as urinary albumin/creatinine ratio. Plasma levels of TNF-α decreased, whereas levels of IL-10 increased in EV-treated patients. Renal biopsy findings 3 months after intervention revealed that EV-treated kidneys showed upregulated expression of cell regeneration and differentiation markers. Importantly, participants did not experience any significant adverse events during or after EV therapy throughout the study period. Therefore, this study suggests that MSC-derived EV therapy is safe and can ameliorate renal inflammation and improve function in patients with CKD. Nevertheless, future long-term follow-up clinical studies need to confirm the persistence of the beneficial effects of this approach in patients with CKD.

Furthermore, significant translational challenges need to be faced before adopting MSC-derived EVs as a useful therapy for AKI and CKD (Table 7). Theoretically, cell-free therapies such as EVs might offer superior advantages over delivery of their parent MSCs in terms of safety. EVs are small particles with no proliferative capacity. Being acellular, EVs should be exempted from adverse effects. Unlike MSCs, EVs can be stored for a long time, allowing their use as “off the shelf” products. Nevertheless, long-term follow-up studies for closely monitoring EVs are needed to determine their safety.

**Table 7:** Challenges for clinical application of MSC-derived EV therapy for renal disease

Challenges	Explanation	Future directions
EV source, isolation, and storage	• MSCs derived from different sources may release EVs with distinct content and regenerative effects • EV isolation and storage methods may potentially affect EV characteristics	• Compare the renoprotective properties of EVs released from different MSC sources • Methods for EV isolation and storage for future clinical studies
Heterogeneity of EV subpopulations	• Exosomes and microvesicles may exert distinct renoprotective properties	• Determine which EV subpopulations show superior regenerative potential in patients with renal disease
Plasticity of EV cargo	• Modulation of ex vivo culture conditions might alter the transcriptional and protein signatures of EVs and potentiate their renoprotective effects	• Identify optimal preconditioning maneuvers
Effect of cardiovascular risk factors on EVs	• Cardiovascular comorbidities are common among patients with renal disease and may limit their regenerative potential • May limit autologous use	• Determine the efficacy of MSC-derived EVs in patients with comorbidities
Fate and engraftment	• Relatively small amounts of EVs are detected in the kidneys after systemic administration • Current detection methods often fail to identify engraftment into renal cell types and monitor the fate of MSC-derived EVs, possibly due to their small size	• Unlike MSCs, EVs cannot proliferate • Might be promptly removed by immune cells • Need to develop tools to target EVs to the kidneys • Need methods to better assess engraftment, survival, and function of MSC-derived EVs
Safety and long-term effects	• EVs modulate the transcriptional and translational machinery of recipient cells • Although MSCs are generally safe, long-term benefits and side effects of exogenous EVs have not been adequately explored	• Explore MSC-derived EVs long-term benefits and potential side effects in patients with renal disease
Delivery regimens	• Dose–response relation and optimal intervals between multiple doses of EVs have not been studied in treatment of renal diseases • The best route of delivery might be invasive (intrarenal)	• Future preclinical and clinical studies are needed to define optimal dose regimen in these patients • Development of kidney-targeted EVs may facilitate systemic delivery

According to recent methodological guidelines [62], several methods could be used to isolate EVs which may impact on EV purity, concentration, morphology, size range, and functional activity [63]. EV handling and storage may also affect their concentration, composition, and function [64]. Therefore, additional studies are needed to test whether renal outcomes vary as a function of EV collection, storage, and isolation methods, and optimize standard protocols for clinical studies.

Few studies have tracked the fate of EVs after systemic in vivo administration, but data from IRI [39] and UUO [58] animal models showed that 24 h after infusion EVs primarily engrafted into the damaged kidney and to a lesser extent in the non-affected kidney [40]. The majority of EVs were taken up by renal tubular epithelial cells (RTECs) and peritubular capillaries [39, 58], but some were identified in glomeruli [33]. In our MetS + RVD model, EV retention was higher in post-stenotic kidney than contralateral kidneys, and EVs engrafted tubular cells and macrophages 4 weeks after administration [55]. This suggests enhanced tissue uptake of EVs under stressful conditions, which may be mediated by infiltrated immune cells or altered expression of surface markers on parenchymal cells. EVs were also observed in the heart, and in large quantities in the lungs, liver, and spleen. Development of kidney-targeted EVs can facilitate their systemic delivery and enhance their regenerative benefits.

The duration and long-term term effects of MSC-derived EVs are important to consider before moving towards their clinical application. In most experimental studies, follow-up ranged from 1 day to 2 weeks post-injection, and only one study in rats with renal IRI found a lower incidence of CKD 6 months after EV therapy [33]. It is clear that EVs can alter transcription profiles in recipient cells, and modulate tissue metabolism and several cellular pathways. Thus, the long-term implications of these post-transcriptional modifications, especially with continuous or repetitive administration of EVs, need to be elucidated. In this respect, their lack of cellular machinery and inability to proliferate in the recipient tissue might limit the duration of their effects and necessitate repeated administration.

There is also uncertainty regarding the optimal dose regimen of MSC-derived EVs, which might influence their capacity to home and engraft damaged cells, and thereby their efficacy for renal repair. Macrophages may promptly target and remove exogenously administered EVs [65], so multiple doses may be needed to achieve and sustain EV-induced renoprotection. A single study found that a multiple dose regimen was superior in decreasing mortality and improving renal function [47]. Administration of larger doses of MSCs was not necessarily associated with better outcomes, and even an inverse dose–response relationship may occur following a high MSC dose [66, 67]. Administration of both low (1 × 10^5^ cells/kg) and high (2.5 × 10^5^ cells/kg) dose of autologous MSCs improved renal blood flow and kidney perfusion to the same magnitude in patients with RVD [22]. However, no study has reported the in vivo efficacy of escalating doses of EVs or determined a threshold dose in experimental renal disease. Therefore, a standard regimen of EV delivery needs to be established in order to test their efficacy in randomized clinical trials. Furthermore, the adequate number of EV injections and the interval between them need to be determined in future studies.

Cardiovascular risk factors may impair the functionality of MSCs and diminish the regenerative benefits of autologous MSC implantation [68]. However, whether EVs isolated from MSCs are also susceptible remains unknown. We have recently found that MetS interferes with the packaging of cargo of porcine adipose tissue MSC-derived EVs, altering the expression of microRNAs that control genes implicated in the development of MetS and its complications [28]. These observations suggest that cardiovascular risk factors may limit the therapeutic efficacy of autologous MSCs and EVs in subjects with coexisting MetS and renal disease. Further preclinical studies and thoughtfully designed and sufficiently powered clinical trials are urgently needed to clarify these uncertainties and overcome the challenges associated with EV therapy in patients with AKI and CKD.

Lastly, emerging evidence suggests that renal cell-derived EVs might also exert tissue protective properties in experimental renal disease. RTECs that line the renal tubules play a crucial role in renal function. Similar to MSCs, RTECs release EVs that serve as intercellular communication messengers and may accelerate renal recovery by eliciting tissue regenerative responses. RTEC-derived EVs similarly contain a rich cargo of mRNAs, microRNAs, and proteins that transmit regenerative signals. TGF-β1-treated RTECs release multiple EVs containing microRNA-21 that enhance PTEN-Akt signaling, which modulates several important biological processes [69]. However, EVs released by injured RTECs also contain TGF-β1 mRNA and microRNAs that activate fibroblasts, and their co-incubation with them promoted collagen production [70]. Speculatively, this function might be related to the injury resolution phase. Unfortunately, none of these studies tested the in vivo protective effects of RTEC-derived EVs.

More recently, intravenous administration of EVs derived from RTECs in rats with renal IRI improved the renal microvasculature and decreased tubular damage and fibrosis [71]. EVs from hypoxia preconditioned RTECs were more effective compared to those obtained from normoxic cells, possibly due to their inhibitory effects on apoptosis following ATP depletion [72]. Fibroblast-derived EVs failed to ameliorate kidney damage in glycerol-induced AKI [48], suggesting that EV-induced renoprotection depends on their cellular source. Therefore, in vitro modifications of RTECs may enhance the protective properties of their daughter EVs. Future studies are needed to confirm these findings and compare the renoprotective potential of MSC- with non-MSC-derived EVs.

## Conclusions

**Fig. 2:**
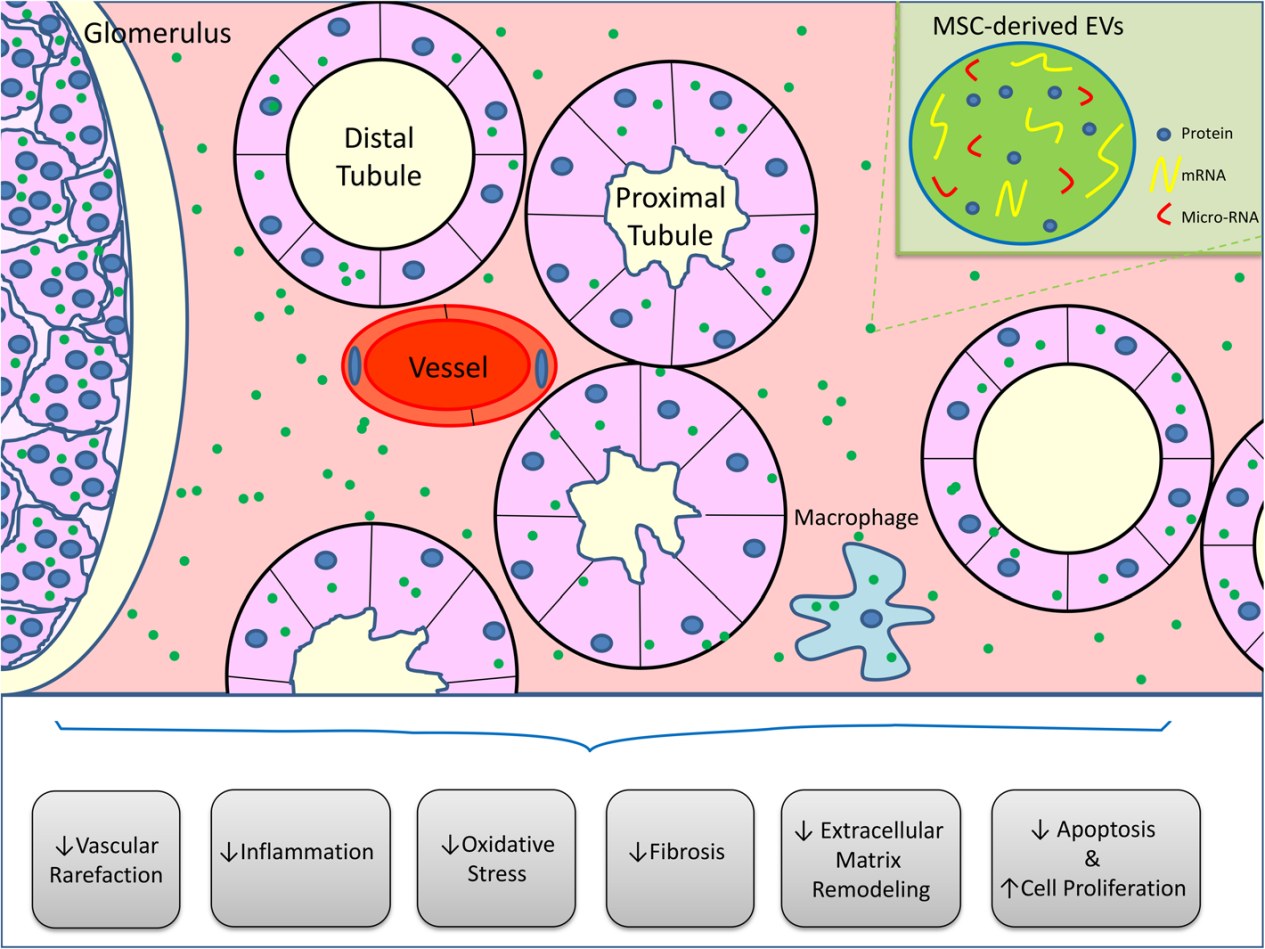
Mesenchymal stem cell (*MSC*)-derived extracellular vesicles (*EVs*) are taken up by renal proximal and distal tubular cells, macrophages, and endothelial cells. MSC-derived EVs transfer their protein, mRNA, and microRNA content into recipient cells. This in turn modulates several pathways involved in the pathophysiology of renal disease, including vascular rarefaction, inflammation, oxidative stress, fibrosis, extracellular matrix remodeling, apoptosis, and cell proliferation. This figure is original for this article

AKI and CKD remain global public health challenges, associated with an increased risk for progression to ESRD and cardiovascular complications. Several characteristics of MSCs tested pre-clinically make them attractive to preserve the kidney suffering from AKI and CKD. There are currently several ongoing or completed clinical trials using MSCs for a wide range of renal diseases and preliminary results suggest that MSCs are safe, well tolerated, and effectively ameliorate renal pathology. MSCs exert their reparative effects by releasing EVs, and recent studies in experimental models of AKI and CKD have shown that MSC-derived EVs offer an effective modern treatment option for these patients. MSC-derived EVs contain genetic and protein material that upon transferring to recipient cells can activate several repair mechanisms to ameliorate renal injury (Fig. 2). Furthermore, these particles offer some exciting advantages over MSCs. However, clinical data are limited and several challenges need to be addressed as we move towards clinical translation. To date, the primary uncertainties for MSC-derived EV therapy for renal disease include insufficient scientific data to support their safety, and the need to identify the most appropriate EV cellular source, isolation method, and dose regimen, and to assess the impact of co-morbidities on their cargo and renoprotective effect. Alternatively, RTEC-derived EVs may also contribute to cellular repair in AKI and CKD, but the beneficial effects of this approach in patients with CKD remain unknown. Therefore, further basic and translational studies need to continue exploring the potential therapeutic applications of MSC-derived and renal cell-derived EVs for AKI and CKD.

## Abbreviations

AKI: Acute kidney injury
BUN: Blood urea nitrogen
CKD: Chronic kidney disease
CXCL1: C-X-C motif ligand 1
DIN: Drug-induced nephropathy
eGFR: estimated glomerular filtration rate
EMT: Epithelial to mesenchymal transition
ESRD: End stage renal disease
EV: Extracellular vesicle
GFR: Glomerular filtration arte
IGF: Insulin-like growth factor
IL: Interleukin
IRI: Ischemia reperfusion injury
MetS: Metabolic syndrome
MSC: Mesenchymal stem cell
RBF: Renal blood flow
ROS: Reactive oxygen species
RTEC: Renal tubular epithelial cell
RVD: Renovascular disease
STN: Subtotal nephrectomy
TGF: Transforming growth factor
TNF-α: Tumor necrosis factor-alpha
UUO: unilateral ureteral obstruction
VEGF: Vascular endothelial growth factor.
